# Origin of the Domestication of the Horse*Read before the twenty-fifth annual session of the U. S. Veterinary Medical Association at New York, Sept. 18, 1888.

**Published:** 1888-10

**Authors:** Rush Shippen Huidekoper

**Affiliations:** Vet. (Alfort)


### ART. XXII.—ORIGIN OF THE DOMESTICATION OF THE HORSE.*

BY RUSH SHIPPEN HUIDEKOPER, M.D., VET. (ALFORT.)

*Bibliography.*—Piétrement, Milne-Edwards, Sir Charles Lyell, Sanson, Ephrem Houël, Emile Burnouf, Rodier, Pictet, Max Müller, Mariette Bey, Layard, et al.

*Etymology of terms for horse.*—The word *horse* is derived from the Sanscrit word Rasa—a sentiment or passion.

*Rasika*—A horse, *i.e.*, a sensible, ardent or intelligent animal; used also for the elephant.

*Hros* —Ancient Saxon and Scandinavian.

*Synonyms.*—*Ross*—German.

*Ors*—Dutch and Swedish.

*Rosse*—French. } A sorry jade.

*Rozza*—Italian. }

The technical term, EQUUS, is derived from the Zend root.

*Açu*—rapid

*Açpa*—A courser; a horse.

*Asp* or *Ash*—Persian.

*Ikkos*—Eolian, which degenerated into *ippos* in the later Greek.

Our adjective, *cavalry*, derived directly from the French, cheval, is found in the

Sanscrit—Tchapala.
Kavi—Kapala.
Irish—Capall, capuill.
Cornish—Kevil.
Welsh—Ceffyl.
Slave—Kobyla.
Latin—Caballus.
Persian—Kawal.
Arab—Chabal.

* Read before the twenty-fifth annual session of the U. S. Veterinary Medical Association at New York, Sept. 18, 1888.

In taking up a rapid sketch of the origin of the domestication of the horse, and the manner in which this useful, or rather absolutely indispensible animal, was first thrown with man, subdued by him and made subservient to his needs, we must first review for a moment the place which he holds in the animal kingdom, and the geographical area in which he was found at the time of the first appearance of man, at which time both animals were in an absolute state of nature.

The Perissodactyla or odd toed animals, had their common ancestor among the earliest mammals, at a time when the Allegheny mountains, and the Black Forest of Germany had the luxuriant flora and beautiful climate of Florida, in the little harmless animal known as the Acerotherium. From this progenitor the cousins of the horse, the rhinoceros and the tapir developed rapidly, and reaching their more simple anatomical form and their lesser intellectual condition at an early period, have remained until the present day mere wild beasts. The remaining branch of the family found themselves in the Eocene period of the tertiary age, well scattered over the face of the earth as a small animal, about the size of a fox, but with, however, the head and body characteristics of the horse of to-day. In the Americas the *Eohippus* found a home in the lowlands, he had forty-four teeth, seven molars in each arch, without cement, tusk teeth, a complete set of digits on the fore feet and four digits with an aborting fifth on the hind ones. The *Orohippus* wandered on the mountains, differing from the other member of the family only in the more rudimentary development of the lateral digits of the hind feet. The Paleotherium, which resembled the mountain American variety, was found in both high and low lands in France, Spain, Greece and in Asia as far as India.

In the Myocene, these animals had developed to the size of a sheep. Their fifth digit was aborted, the lateral ones were rudimentary, their radius and cubitus had become united, they still retained forty-four teeth, the seven molars in each arch still separated into two groups of four anterior and three posterior, and these were yet devoid of cement. The *Eohippus* and *Orohippus* of America were succeeded by the *Mesohippus* and *Miohippus,* while the Paleotherium of Europe takes the name of Anchitherium. In the *Pliocene* the subdivisions of the family were more numerous, and the animal had reached the size of an ass. The seven molars of each arch united into a single line and became covered with cement. In the permanent dentition there were frequently but six instead of seven teeth. The *Protohippus, Parahippus,* Hipparion or Hippotherium and *Pliohippus* and numerous other localized divisions of the family only differed from each other in the more solid fusion of the cubitus and radius and in the gradual more rudimentary form of the lateral digits, until the animals stood upon a single toe, the lateral metacarpals and metatarsals having aborted into the splint bones of the horse of to-day, the lateral digits having become the single ergot behind the fetlock. The six molars with cement became the rule, while the seventh molar, which is the "wolf tooth" of our own horses, was the exception. With this development at the end of the tertiary and the commencement of the quartenary periods, the *Genus Equus* abounded all over the world; it survived the glacial periods in Europe and Asia, but disappeared entirely from Africa and the Americas, to reappear in the former with the invasions of the Hyksos, and in the latter with the advent of the Spaniards. The Equides, also known to the earlier Latin writers and until 1693 A. D. as "*solidipedes,*" and since then by the name given them by John Ray of "*solipeds,*" formed numerous families at the beginning of the quartenary age, but soon afterwards the Genus Equus was reduced into four distinct tribes or species. These are the *Equus caballus,* or the horse : the *Equus asinus,* or the ass; the *Equus hemionus,* or the hemion, and the *Equus zebrus,* or the zebra. The latter, the African representative, can be subjugated but never domesticated. The Hemion of Asia is capable only of a semi-domestication. It has the endurance of the ass and the agility of the horse, but is always treacherous ; except for this last quality it would be a most useful animal. The horse and the ass alone interest us as domestic animals. The *Equus caballus,* or the horse, at an early period, as shown by the cranial types in early fossil deposits, was again subdivided into species, of which eight now remain, viz.:

*E. C. aryanus*—improperly known as the Arab.
*E. C. mongolicus*—the so-called African Arab.
*E. C. hibernicus*—the Irish cob.
*E. C. britannicus* the English shire horse.
*E. C. frisius*—the Dutch horse and Clydesdale.
*E. C. germanicus*—the Danish and Luxembourg horse.
*E. C. belgicus*—the Ardenner post horse.
*E. C. sequanus*—the Percheron.

A ninth small variety still existed in the lower Danubian countries at the time of the voyages of Herodotus and Strabo, but owing to the custom of castration and the superior advantages of other races it has entirely disappeared.

The *Equus Asinus*, or the ass, is subdivided into the *E. A. orientalis*, or *africanus*, or Egyptian ass, and the *E. A. occidentalis*, or European or Spanish ass.

In the fossil deposits of Aurignac, in the Haute Garonne, are found the first traces of the horse in connection with man. Here, with human bones and stone implements of the rudest sort, are found the remains of nine species of carnivora and ten species of herbivora ; among them the great bear (*ursus spalœus*), the great cat (*felis spalœa*) the hyæna, the mammouth, the rhinoceros, the great deer, the reindeer, the auroch and the *horse*.

The bones remaining of these animals are only the long ones, all broken in the centre and bearing traces of the use of the chipped stone implements used to split them open. No vertebræ except the two nearest the head, and no ribs are found. Heads with crushed cranial bones and inferior maxillary bones exist in numbers. In the deposits of Lourds, Milne Edwards found the bones of man, chipped stone instruments, the bones of the horse, stag, chamois, aurochs and those of other animals ; phalanges, crushed long bones, heads with broken crania, atlases and axis existed in quantity, but no other bones were found ; the bone around the roots of the stags' horns and the cannon bones of all animals bore traces of the blunt instruments used to remove the hides.

The troglodytes hunted these animals for food ; they found the carcasses too large to transport to their caves, so removed the legs for meat, the heads for the brains, and, after feasting on the flesh, split open the long bones for the marrow. They may also have employed the brains and marrow for tanning the hides, as is done by the Pallas and by some tribes on the coast of the Arctic to-day. No trace of the dog is to be found in these earlier remains.

In the Kjökkenmöddings of Denmark we also find the remains of the horse, and in the later tumuli of the same region these remains are found in greater quantities, and with those of the dog more intimately associated with those of man and the early stone implements of the latter.

In the Bronze Age we find the traces of the horse much more intimately associated with the remains of man. In the second lacustral period of Switzerland, in the Dolmens of Denmark and in the Bohemian homes of the human race, surrounded by castings and hammered implements of bronze, are found the remains of the horse, the dog and the ox in such numbers and in such predominance over the bones of other animals, that we are warranted in believing that man had found an easier existence in domesticating the young and wounded animals of these species, and in having them for food and raiment always at hand, than he had before held, in being obliged to make long journeys through the wilds, to slaughter absolutely wild animals, and to struggle back to his cave or hut with the quarter and hide on his shoulders.

We may assume then, that the first domestication of animals was made in the Bronze Age. But at this period we find the evidences of man altered. The Dolico-cephalic races of Cro-magnon and of Canstadt have been replaced by the Brachy-cephalic tribes of men, who are gradually moving from the Orient into the fertile valleys of Central and Western Europe. The appearance of the more perfected instruments of domestic use, and the close association of the domestic animals with their human masters correspond with the earliest migrations of the more intellectual and advanced members of the human family from the East. Intermingled with the traces of the domestication of the indigenous races of the horse and the ox of Europe, are to be found the remains of the Aryan horse of Asia. Evidently as civilization moved slowly from the Orient, a sufficient number of animals accompanied it to supply the absolute needs of the pre-historic nomads; but, as the troglodytes were conquered, and, as the population increased, the indigenous animals were domesticated, and the native people were taught the art of a primitive agricultural life by their invaders.

The first domestication of our animals was a necessity imposed upon pre-historic man, when his awakening intelligence taught him to care for the aged and the infirm. When these and their increasing families could no longer depend upon the precarious chances of the chase, the nobler race of man found that he must hoard a supply at his dwelling. He found that he must foresee the day of his own decline. The stronger discovered that it was easier to live by the labor of the weaker than to work himself. He became an autocrat—a rudimentary civilian—and counted his standing *per caput*, by the number of heads of less intelligent human beings, horses and cattle which he could command, a capital which has remained to us, until the Nineteenth Century of the Christian era, although sometimes reckoned to-day in dollars and cents.

Such is the primitive relation of the horse to man. We must now pass over an indefinite period to a more accurate association of the two.

In the Balkan Mountains in Asia, where the great rivers flow to all parts of the old world—about the present location of Punjab, the pass which separates the covetous eyes of Russia, and of England through India to-day—the first truly civilized people, of whom we have any fairly evident history, developed. They built cities, employed metals, possessed animals in domestication, learned to express their ideas in cuniform inscriptions, multiplied in numbers, and as a result were forced to seek a larger area and greater supply for their needs.

The Aryans, as these people are called, commenced their migrations, taking with them the animals which had already become a necessity, at a period fixed by Emile Burnouf at a little more than 19,000 years before the Christian era. A few thousand years later they had divided into the great Hindoo race, which had followed the bed of the Ganges, and the Iranians or Aryo-Persians who travelled to the West. By this time their cuniform literature was well advanced. Their religious writings, the *Rig-veda*, the *Zend Avesta* and Vendidad, based upon common law and justice to communities, like those of all later nations, were made, and in them we find the first written history of the horse. In these we find the word açva, açpa or asp indicating the horse. In the writings of the times preceeding the Zoroaster and the fall of Djamschid, 1000 years B. C., we find that the rulers and great men were designated with names combining this root of "*asp*" indicating the possession of of horses—for example, Shedasp and Gnenasp, both rulers of Irania.

The few following quotations from the large number which are contained in the religious works will serve for proof :

*Açwameda, or Sacrifice of the Horse.*

"Here are my prayers, * * * that the horse with elongated croup will happily fulfill the hopes of the Gods. * * * *

"It is by happy ways that thou shall go to the Gods. To carry thee thou hast the two racers, the two antelopes and the chariot drawn by an ass. * * * *

"The ass cuts the *thirty-four* ribs of the rapid horse. Leave entire the outer parts that each member shall be properly prepared.

"If those who see the horse cooked say 'it smells good,' cut them a piece. Grant the demand of whomsoever wishes of this flesh.

"May the horse procure us numbers of cattle, of good horses, of children, of great wealth. Thou who art pure and strong make us strong and pure. May the horse, honored by the holoclast, give us strength."

"O Horse, after then the mortals and their chariots, and then cattle, and the happiness of their young girls. All living beings search thy favor. The gods would equal thy force. * * * *

"His mane is of gold: his feet as rapid as a thought. *Indra* has descended. The gods have come to celebrate the Holoclast of him who first has used the horse.

"These princes have given me fifty horses, and I have paid for this present in hymns.

* * * "A pious king, prudent and generous Tryaruna son of Trioris-na has made me rich; he has given me two oxen harnassed to a chariot, with ten thousand cows. May he remember thou.

"He has given me twenty cows and two horses drawing a precious load—a proof of the value of the horse and that the ox was the beast of burden. May the racers covered with gold given me by the generous Trasadasyn, son of Purukutis, may the ten *white horses* of Girixita draw me to the assembly of the sacrifices. I have received from Vidata * * * strong and magnificent horses of a red color.

"Young and magnificent, superb and fertile, burdened by its rich banks, it sees on its sides good horses, rapid chariots, flocks with silken wool."

We have in the above, proof that the horse was eaten and that he was used for sacrifices to the gods. From the existence of variable colors we have proof of the already ancient domestication of the animal. From the reference to the 34 ribs we have support in proof of the existence of a Mongolian horse which had two less ribs than other species. We have, lastly, proof that the Vedic-Aryans or Indo-Iranians used the horse at the commencement of the Vedic age, that is 19,337 years before the Christian era.

In the Vendidad, Chap. V., verse 152: The milk of a mare, fresh and pure is one of the foods prescribed for a woman who has just had a still-born child.

In the *Rig-veda* the laws of *Manon*, anterior to the establishment of the Hindoo castes, impose a penalty for the killing of a horse; they decide the ownership of a foal when the mare is the property of one and the horse of another. The sacrifice of the horse to the gods is regulated. Description is given of horses, mounted, in chariots, as pack animals, in races and of their being of various colors.

Greece did not possess the horse at the commencement of the Heroic Age, though Homer describes the plains as covered with horses at the time of the seige of Troy (1219 years B. C.)

According to legend, Neptune gave the horse to Athens at the founding of the city. The earliest Phenœcian navigators who had reached the shores of the Mediterranean as wandering horsemen, and became sailors to satisfy their greed for travel and conquest, placed horses heads on the prows of their vessels. Neptune, God of the sea, was also named Hippius, and Minerva, goddess of war, received the name of Hippia (*ippos*, a horse.) The horse was plentiful in Assyria at the time of the extension of Nineveh by Ninus (3274 B. C.), according to Diodorus.

The excavations in the valleys of the Tigris and the Euphrates, and the translations of the cuneiform inscriptions confirm the accounts of Diodorus as to the utilization of the horse by the Assyrians. The researches of Layard at Nineveh show that the horse was used for agriculture as well as for war.

The earliest Greek warriors derived many of their names from the horse, as Hippocoon, Hippodamus, Hippolochus, Hippotheos, who were at the siege of Troy.

The first horse of Achilles and of Hector are described "Xanthe," or yellow-red, indicating that the animal was a sorrel. Red (bay), sorrel, white and dun horses are noted. In the Iliad XXIII., we learn that horses were castrated on the 16th day of the month, hogs and the ox on the 8th, mules on the 12th. Xenophon, who served as a youth in the army of Cyrus the younger, wrote at length on equitation and cavalry. He gives rules for castration and for the subjugation of vicious horses and kickers. He gives formula for hardening horses hoofs, as shoeing was then unknown. He describes the perfect horse as one with an open eye, small ears wide apart at the base, large chest, rounded ribs, large short loins and large muscled croup. In the army of *Xerxes* the Magi sacrificed white horses to future success.

Under *Cyrus* horses were sacrificed to the Sun, and bulls to Jupiter. Races were run mounted and in chariots.

Darius placed an impost on the Cicilians of 360 white horses a year.

He fought with a cavalry which is variously given from 41,000 to 200,000. Under *Alexander the Great* the troops refused to move as the "*feet* of the horses were *worn* by continuous marching." Shoeing was unknown in the eight conquered countries of Asia Minor, Syria, Egypt, Mesopotamia, Hyrcania, Bactriania, Sogdiana, Persia and India.

The horse was only used in *Egypt* at a relatively late period. It did not exist there during the first historic periods. The Egyptians did not possess horses during their first invasions of Asia under Sesostris (3433–3495 B. C., and 12th Dynasty.) He was imported into Egypt and naturalized at the time of the invasions by the Hyksos (2898 to 1945 B. C.) Under Rameses the Great, the Egyptians, however, had large bodies of cavalry.

Although *Arabia* is to the vulgar mind the country of the origin of the horse, we find that this animal was only introduced into the peninsula at a very late date.

Herodotus describes the Arabs and Arabia, the two races of sheep, cattle, asses and goats, the products of the country, the presents given by the Arabians to the Kings of Persia, but he makes no mention of the horse. In his accurate descriptions of other countries, however, he describes in detail those of Babylon, of Cicilia, of Media, Thessaly and India. He details the horses used in the Olympean games, which were won by mares. He describes the Army of Xerxes, but of the Arab contingent mentions only their costume and arms. He says that the "Lybians fought in chariots of war. * * * The Caspirians, the Paricanians, the Arabs, cavalry, equiped like the infantry, except that the last were mounted on camels which were equal to the horses in speed. These were the only nations which furnished cavalry, the number of horses was eighty thousand, without the camels and chariots. They were organized by nations, the Arabs the last, as the horses were afraid of the camels."

*Strabo*, an intimate friend of the Governor of Egypt,

Aelius Gallus, who undertook a disastrous expedition into Arabia, describes the Arabs as a rich commercial people, describes their possessions, says they are great warriors; and that in their caravans they employ only camels.

Of the north of Arabia, Strabo says they are rich in the products of agriculture and cattle, but are poor in horses, mules and hogs.

*Arrian* describes the thousands of horses of the Army of Alexander, he gives detailed account of the Arabs and the extent of their country, but does not mention the horse.

The Roman Authors do not mention the horse of Arabia before the 4th century. Pliny gives a lengthy description of horses of different countries but does not include Arabia.

The first note of the horse in Arabia is by Ammian Marcellus, a Roman officer of Greek origin in 353, A. D.

The Chinese possessed the horse at a very early period and their chronology which antedates in accuracy that of any other nation and becomes absolute after the year 2698 B. C., when under the reign of Hoang Fi, a tribunal of history was established, includes valuable information regarding five other domestic animals.

These were all introduced into China during the conquest of the Eastern provinces by Fo-hi the 17th King of the 9th Dynasty, 3468 before the Christian Era. In the reign of Chin Hong, 3218, B. C., the cultivation of the silk worm was added to the agricultural industries. The Chinese word of "*Ma*" for the horse is found combined into a title of Se-ma the Chief of the Horse, in 1115, B. C., but horses were then still few and were used for Royal gifts.

The Royal Household had 1000 chariots of 16 horses each. A Prince had 100 chariots of 16 horses, to which number he was limited by law. Every 800 families of the people were obliged to furnish one chariot and sixteen horses with three armed captains and twenty-two footmen.

The *Hebrews* offer a curious exception to other nations, who, as we have just seen, employed the horse almost universally in prehistoric times. Among these people the horse only came into use with the reign of the kings. Throughout all the wanderings of the earlier people, no horses were used by them. Under the rule of the Judges, although the increasing luxury and wealth of the people tempted them to emulate their neighbors in the possession of horses for pageants and for cavalry for their armies, the order of Moses forbidding the use of the animal was still regarded. The great legislator of the world wanted his people to be a just and humble one. He wanted them to live among themselves honoring their own families and not annoying their neighbors nor their neighbors' goods. He foresaw that the horse was an article of pomp, of vanity, and of war. Its use tempted the owners to long journeys, the possession of cavalry stimulated the people of the day to conquest. He allowed the use of the ass but forbid that of the horse. Before the voyage of Joseph into Egypt there is but a single mention of the horse in Genesis, although there is frequent mention of numbers of the other domestic animals in the description of the riches of some of the great personages.

The only passages of Genesis which refer to horses or chariots are in reference to the Egyptians, with whom the Jews had intercourse.

We find various animals used for sacrifice by the Hebrews, but never the horse, which among all other nations was so frequently used for this purpose.

We find throughout all the wars of King David that the Hebrews were thrown into conflicts with and captured large bodies of cavalry and war chariots, but no mention is made of the disposition of them. In King Solomon's time, ten centuries before Christ, the magnificence of parade and pageant could no longer regard the religious warning, and we find the great monarch not only using numbers of horses but taking advantage of his position as son-in-law to the Pharaoh of Egypt, not to pay the export duty levied on horses and becoming a great dealer of his country, an example now emulated by many of the race, as the largest horse dealers of New York and all the great European cities to-day are Hebrews.

Job possessed only cattle, but had evidently seen or heard of the war horse, which he evidently greatly admired as an emblem of strength.*

*America.*

We have seen that at the end of the tertiary age the Americas were rich in the equides, but they suddenly disappeared at about the time of the Glaciers. The only domestic animal found was the dog. The horse reappeared in this country in 1516 in the island of Bastimentos, and a year later in Mexico. Under Cortez about fifteen horses were landed, of which we have the minute description as to color and qualities. We find the descendants of escaped horses which people the West and South America, in a state of freedom, with the same characteristics of cranium and croup as is found in the *E. C. mongolicus* and its variety, the Spanish Barb.

